# The Lived Experience of Employed Mothers Transitioning Back to the Workplace While Breastfeeding

**DOI:** 10.3390/healthcare14213766

**Published:** 2026-11-04

**Authors:** Sumathi Robert Shanmugam, Nojoud Abdullah Alrashidi, Grace Ann Lim Lagura, Samah Elawady Bassam, Ohood Ali Alkaabi, Joyce Buta, Zainab Alharbi, Lea Dando, Maysa Mohsen, Reynita Saguban, Evalynn Rondilla

**Affiliations:** 1 Department of Maternity and Pediatric Nursing, Princess Nourah Bint Abdulrahman University, Riyadh City 11671, Saudi Arabia; srshanmugam@pnu.edu.sa; 2 Maternal and Child Nursing, College of Nursing, University of Hail, Hail City 55476, Saudi Arabia; 3 Department of Maternal & Child Health Nursing, College of Nursing, Qassim University, Buraidah 51452, Saudi Arabia; 4 Department of Pediatric Nursing, Faculty of Nursing, Zagazig University, Zagazig 44519, Egypt; 5 Department of Nursing, University of Bisha Al Namas, Al-Namas 61922, Saudi Arabia; 6 Fundamental of Nursing Department, College of Nursing, King Khalid University, Abha 61421, Saudi Arabia; 7 Psychiatric Mental Health Nursing, College of Nursing, University of Hail, Hail City 55424, Saudi Arabia; 8 Department of Maternal and Child Nursing, College of Nursing, University of Hail, Hail City 81451, Saudi Arabia

**Keywords:** breastfeeding, lactation, maternity leave, mothers, workplace

## Abstract

**Background:** Returning to work after childbirth is one of the major factors leading to early cessation of breastfeeding. Article 154 of the Saudi Labor Law gives employed nursing mothers the right to a daily breastfeeding break. However, little information is available regarding employed mothers’ experiences of returning to work while breastfeeding. To date, no qualitative studies have examined this transition experience among employed mothers in Hail City. **Methods:** This study employed a qualitative descriptive phenomenological design based on Husserl’s transcendental phenomenology and Colaizzi’s seven-step model. The researchers recruited 13 breastfeeding mothers (aged 27 to 32 years), who worked in various sectors such as government, education, healthcare, and private sectors, via purposeful and snowballing methods. Interviews were carried out face-to-face in Arabic, lasted approximately 30 to 50 min, and occurred one to eight months after the mothers returned to work. Saturation was reached when three consecutive interviews did not produce any new code or theme. All interviews were audiotaped and transcribed verbatim in Arabic and translated into English, with the translation verified by a bilingual researcher who cross-checked a random sample of transcripts against the original Arabic audio recordings to ensure conceptual and linguistic accuracy. Data analysis was completed using Colaizzi’s seven steps, with NVivo software used only to manage the data. Trustworthiness was established using Lincoln and Guba’s criteria, including member checking, triangulation of sources, thick description, maintenance of an audit trail, and reflexive bracketing. Five of the 13 mothers reviewed and confirmed the summary of findings during member checking. **Results:** Five themes described the essential structure of the experience: (1) return anticipation, preparation, and quiet resolve; (2) the workplace as an unaccommodating space, with twelve of thirteen mothers lacking a lactation room, and most reporting no protected time and no safe milk storage; (3) the gulf between the law on paper and the law in practice: only two mothers knew of their Article 154 entitlement, and in participants’ accounts access rested on informal arrangements with supervisors rather than on stated workplace policy; (4) straddling two worlds, the guilt, anxiety, and divided identity of performing maternal and professional roles at the same time; and (5) circles of support, with family help, coworker solidarity, and personal determination described as key facilitators. Taken together, the accounts of the participants in this sample pointed to a fundamental structure of solitary negotiation. Mothers continued breastfeeding largely through personal and family effort, with little institutional support available to them. **Conclusions:** In this sample of employed mothers in Hail City, breastfeeding after return to work was sustained largely through personal and family effort rather than enforceable rights. These findings are specific to one city and one group of mothers, so they may not apply to other settings. They still point to the possible value of enforcing and raising awareness of Article 154, providing workplace lactation facilities, training supervisors, and offering anticipatory breastfeeding counseling during maternity leave.

## 1. Introduction

Breastfeeding is widely recognized as the optimal source of nutrition for infants, and the World Health Organization (WHO) recommends exclusive breastfeeding for the first six months of life, yet many countries still fall short of this standard [1]. It has been estimated that optimal breastfeeding could prevent 823,000 child deaths annually [2]. Cognitive losses from failure to breastfeed have been estimated to cost the global economy $302 billion annually [3]. In addition to benefits for infant mortality and cognitive development, breastfeeding has long-term health benefits for both the child and the mother. These include prevention of infectious and non-infectious diseases and a lower risk of breast and ovarian cancer [2]. A woman’s decision to return to work remains a major factor associated with how quickly she stops breastfeeding, though it operates alongside multiple other individual, family, and workplace influences. An analysis of national policies in 182 countries found that guarantees of paid breastfeeding breaks for at least six months were associated with an 8.86 percentage-point higher rate of exclusive breastfeeding [4].

Conversely, maternity leave of six weeks or less has been associated with fourfold higher odds of failing to establish breastfeeding. Among mothers who did establish breastfeeding, short leave was linked to more than threefold higher risk of early cessation [5]. A woman’s ability to continue breastfeeding upon returning to work is often contingent on her employer offering adequate break time and a private place to pump and store milk; without such supports, sustaining lactation after returning to work is very difficult [6]. Although workplace support is relatively uncommon, when it does occur it comes most often from family and partners, and only rarely from the employer organization. In Australia, 60% of women returning to work after maternity leave intended to continue breastfeeding their babies, yet only 40% were able to do so. Only 13% of these mothers received any support from within the Health Service, and only 6% received any support from Human Resources [7].

These international trends have direct implications for Saudi Arabia’s Vision 2030. Female labor force participation has risen substantially over the past decade, reaching 34.5% among Saudi women in Q2 2025, while the overall labor force participation rate for Saudis and non-Saudis reached 67.1% [8]. The Saudi Labor Law provides formal protections for nursing employees under Article 154. This article allows a daily breastfeeding rest of up to one hour in total [9]. The hour is in addition to the ordinary rest periods granted to all workers. It is counted within actual working hours and does not reduce wages. It should be noted that Article 154 applies to workers covered by the Labor Law, and we identified no published evidence on how it is applied and enforced across public sector, educational, and private employment. At the national level, research documented a steady decline in overall breastfeeding duration in Saudi Arabia over the preceding 25 years. In that review, four of ten studies found that working mothers breastfed less frequently and for shorter durations than non-working mothers [10]. Working mothers in Saudi Arabia face several challenges when trying to continue breastfeeding after returning to work. Early return to work was the most commonly reported barrier to exclusive breastfeeding among working mothers surveyed in Al-Ahsa [11], while working mothers surveyed in Riyadh reported having no designated place to breastfeed or express milk at work [12].

Qualitative research expands our understanding of this situation. Researchers used a phenomenological approach to examine how nine working mothers experienced transitioning back to work at a hospital in Riyadh. They found that the transition was shaped by the availability of space for breastfeeding, flexibility in the job, workplace breastfeeding policies, and supervisor behavior. Most participants discontinued breastfeeding before their infants reached six months. The few who persisted did so through personal determination rather than workplace accommodation [13]. Most of the available Saudi evidence is quantitative and concentrated in one major city and a small region in Riyadh. Also, national systematic review and meta-analysis has since examined Saudi women’s breastfeeding knowledge, attitudes, and practices [14].

To confirm the identified research gap, we searched PubMed, CINAHL, Scopus, and Web of Science in June 2026 using combinations of the terms breastfeeding, lactation, breast milk, return to work, employed, working mothers, workplace, Hail, Saudi Arabia, and qualitative. We also checked the reference lists of the studies we identified. The search identified one qualitative study of working mothers, conducted at a hospital in Riyadh [13]. It also identified two quantitative studies from Hail. One was a cross-sectional study of exclusive breastfeeding prevalence among mothers in Hail [15] and the other was a randomized trial of a nursing education program on breastfeeding self-efficacy among first-time mothers in Hail, conducted by members of our research group [16]. No published qualitative study was identified that examined the experiences of employed mothers in Hail City returning to work while breastfeeding.

The evidence gap is not only geographic. The Riyadh study included hospital employees only [13]. The experiences of teachers, administrators, bank and retail workers, and other employees outside healthcare have not been documented. Hail City also has a different employment structure, with maternal and child health services delivered mainly through primary care clinics rather than large hospitals. These differences may shape how the return-to-work transition is experienced. This research is designed to identify the essential structure of an individual’s experience. It allows researchers to focus on an experience as experienced, without the influence of theory. A grounded theory design would have been preferable had the purpose been to develop a theory explaining breastfeeding behavior in the workplace, and an ethnographic design would have suited a purpose of describing the culture of women breastfeeding at work in a particular workplace over time. A Husserlian descriptive phenomenological research design was chosen because the purpose of the research is to describe an experience that has a definite beginning and end (transition) and describe that lived experience as it is experienced in its essence. It is therefore well-suited to capture the texture of the experience of transitioning back to work after having a child. The aim of this study was to describe the lived experience of employed mothers in Hail City, Saudi Arabia, as they returned to paid work from maternity leave while continuing to breastfeed, within the first 12 months after their return. The research question was the following: What is the lived experience of returning to work while breastfeeding among employed mothers in Hail City?

## 2. Methods

### 2.1. Research Design

This study employed a qualitative, descriptive phenomenological design guided by Husserl’s transcendental phenomenology. Colaizzi’s seven-step method was used for data analysis [17]. Descriptive phenomenology aims to identify the fundamental nature of an experience and to provide a faithful account of how that experience exists [17,18]. The researchers were guided by Husserl’s philosophical approach when applying bracketing, also called epoché. Bracketing was treated as an ongoing effort to notice and hold in check the researchers’ personal beliefs, clinical experience, and previous research findings. The goal was for participants to recount their experiences in their own words, as far as this is humanly possible [19,20]. The research team did not claim to have removed all prior knowledge. Instead, they used reflexive journaling and regular discussion to identify assumptions as they arose and to prevent them from shaping the analysis.

### 2.2. Research Setting and Study Period

This study was conducted in Hail City, the capital of the Hail region in northwestern Saudi Arabia, between 21 July 2026 and 21 August 2026. Hail City is an average-sized city with governmental and non-governmental employment sectors, including education, health care, and private industry. Like many other cities throughout the Kingdom, it has seen increasing female participation in its workforce. Interviews were carried out in private areas selected by each participant for maximum privacy and comfort.

### 2.3. Sample and Sampling

The study population included employed mothers who lived in Hail City, had recently returned to employment after maternity leave, and were either currently breastfeeding or attempting to do so at the time of their return. A combination of purposeful sampling and snowballing was used. First, purposeful sampling was undertaken. The researchers initially contacted potential participants, called seed participants, through maternal and child health contacts in Hail. These included mothers attending maternal and child health clinics at primary care settings who met the inclusion criteria. After each participant was interviewed, she was asked whether she knew an employed breastfeeding mother in her social or professional network. If she could identify someone and gave consent, the researchers contacted that person directly to discuss participation. This snowball process continued until data saturation was reached. No new codes or themes emerged during the 10th interview; three further consecutive interviews (the 11th, 12th, and 13th, bringing the total number of participants to 13) were then conducted, and these produced no new codes or themes, confirming saturation.

Of the 18 mothers approached for participation in the main study, 16 met the study’s inclusion/exclusion criteria and two were ineligible. Three of the 16 eligible mothers refused to participate, citing time commitment and family responsibilities. Thirteen mothers were included in the analysis. The separately conducted pilot interview is not included in these recruitment numbers or in the final analytic sample. Four were recruited directly as seed participants through maternal and child health clinic contacts and nine through snowball referral from a previously interviewed participant. Sample size in qualitative research is generally guided by the concept of information power. The more focused the study question, the more homogeneous the sample, and the stronger the interview dialogue, the fewer participants are needed [21]. Sample size is also guided by saturation rather than by statistical power. Sample sizes of this magnitude have been shown to be adequate for phenomenological studies of specific experiences [22,23].

### 2.4. Inclusion and Exclusion Criteria

The study included employed mothers working in any sector (government, education, healthcare, or private) in Hail City, Saudi Arabia. Mothers were eligible if they had returned to paid employment within the preceding 12 months after maternity leave for a live birth, and if their infant was 12 months of age or younger at the time of the study. Eligible participants were breastfeeding directly or through expressed milk upon returning to work, or had at least attempted to breastfeed after returning. In the final sample of 13, all participants were continuing to breastfeed (directly or through expressed milk) at the time of interview. No enrolled participant had ceased breastfeeding entirely before the interview. They also had to be able to speak and understand Arabic and be willing to participate in a face-to-face interview and give informed consent. Mothers were excluded if they never initiated breastfeeding, did not return to employment after childbirth (for example, those who became full-time homemakers), had a documented medical contraindication to breastfeeding, or if their most recent return to work had occurred more than 12 months before the study.

### 2.5. Data Collection

Data were gathered using semi-structured, in-depth, one-on-one, face-to-face interviews. Interviews were completed in Arabic by two researchers: the second author (Nojoud Alrashidi, PhD, Associate Professor of Maternal and Child Nursing) and the sixth author (Joyce Buta, PhD, Assistant Professor of Maternal and Child Nursing). Both researchers are registered nurses with academic and clinical experience in maternal and child health and breastfeeding support, and both had formal training in, and prior experience of, qualitative research interviewing methods. Both interviewers are women. Neither researcher had previous personal or professional connections with the participants; in the few cases where an indirect connection through the clinics’ referral networks could not be ruled out, that participant’s interview was assigned to the co-researcher with no prior knowledge of her to further reduce the risk of bias due to existing relationships and/or differing levels of power. Because data collection occurred in clinical settings and professionals assisted with recruitment, the researchers used the following procedures to ensure voluntariness. Potential participants were initially introduced to the study by administrative personnel or clinic nurses who were not involved in their direct care. These staff members introduced themselves, briefly explained the purpose of the study, and distributed an information sheet that clearly stated that participation in this study was strictly voluntary and that declining participation, or withdrawing from it, would not result in any adverse effects on the type or quality of medical care or services provided to them or their infants.

An interview guide was developed based on the research question and phenomenology literature used to develop this study. The guide was reviewed for clarity and content by two qualified qualitative researchers. It was then pilot-tested in a separate interview with one eligible mother who was not among the 18 mothers approached for the main study. Her interview was used solely to assess and refine the guide and was not included in the main study dataset or analysis.

These revisions clarified technical language, reordered the more psychologically uncomfortable questions so that they would be asked later in the interview, once the interviewee had had time to become comfortable, and added follow-up questions to help identify potential issues related to time limitations during working hours. The first question within the interview guide was the grand tour question, which read, “Describe your experience of returning to work while continuing to provide breast milk to your infant.” The remainder of the interview guide included questions addressing specific areas of interest, including anticipatory preparation for the end of maternity leave, initial experiences and adjustment to returning to work, utilization and accessibility of the designated nursing hour and the lactation facility, interactions with supervisor(s) and colleagues, sources of support, emotional/physical feelings about the experience and what it meant to the mother as a mother. The full interview guide is available as Supplementary File S1.

Each interview lasted approximately 30 to 50 min and was audio-recorded. Participants provided informed consent for this recording. These audio recordings were supplemented by researcher field notes, which captured the researcher’s reflections as well as nonverbal behavior exhibited by the participant during the interview, including contextual factors. These interviews took place between one and eight months after each participant returned to work, in order to allow them enough time to adjust but still be able to remember clearly how they experienced returning to work. At the conclusion of each interview, a short demographic questionnaire was administered. This included items such as age, education, employment, industry/sector, number of children, child age at time of interview, length of maternity leave, and whether or not they were breastfeeding at the time of return to work.

### 2.6. Data Analysis

Following the interviews, each was transcribed verbatim in Arabic. These Arabic transcripts faithfully reflected each participant’s speech and served as the basis for the translation and verification processes. Next, the Arabic transcripts were translated into English by one of the researchers, who was fluent in both languages. The translations were then independently checked for accuracy by a second bilingual nurse researcher using independent cross-checks of the translated transcripts against the original Arabic tapes as well as selected back-translation checks. Any discrepancies were reviewed and resolved through discussion. Colaizzi’s seven steps for descriptive phenomenology were then applied to the verified English translations [17]. Because both analysts were bilingual, they referred back to the original Arabic transcripts and audio recordings throughout the analysis whenever the meaning of a statement was ambiguous, and every quotation reported in this manuscript was checked against the Arabic original. First, the researchers engaged in a familiarization process. They reviewed and re-reviewed the transcripts until they could capture each participant’s whole story. Then, they identified specific statements that represented the participant’s transition back to work while breastfeeding. They derived meaning from these statements and recorded a formulated meaning for each. From there, they organized the formulated meanings into themes. These themes were brought together into a comprehensive description of the lived experience of transitioning back to work while breastfeeding. Finally, they were synthesized into a structural essence representing the core characteristics of the experience. To validate the findings, the researchers returned to the participants to check that the findings represented their actual experiences.

The themes and coding of this study were developed by the first and second authors. Both analysts coded their first three sets of transcripts independently and then compared their coding to develop a common set of codes. The analysts developed code definitions by discussing and resolving differences in their coding. After developing those common definitions, both analysts continued to use them to develop a common coding framework for the remaining transcripts. Any remaining disagreements were resolved through further discussion, consultation with other team members, or an independent outside qualitative researcher consulted as part of peer review. Researchers created analytic memos that documented every decision made during the interpretation of results. An audit trail documenting changes in coding was maintained. The code definitions and analytic memos form part of this audit trail and are available on request.

NVivo (version 15) qualitative data-management software was used only to store and retrieve the data and to organize the coding; all analytic steps were performed manually by the researchers.

### 2.7. Trustworthiness

The trustworthiness of the study was addressed according to the four criteria of Lincoln and Guba [24]. Credibility was established throughout the study by consistently engaging with the data, member-checking, and triangulation of sources. Because participants were recruited through two distinct routes (maternal and child health clinic contacts and snowball referral) and spanned four employment sectors (government, education, healthcare, and private), convergence of accounts across these different workplace contexts could be examined. Member-checking occurred at step 7 of Colaizzi’s methodology. The mothers involved in the study were provided with a synthesized summary of the major findings, central theme, and essential structure (essence) of their experience; it was written in plain, unscientific language in order to be understandable by them. Of the 13 mothers included in the study, five participated in the member-checking process and were contacted via follow-up telephone call or encrypted messaging (WhatsApp), based on their preference. Upon review of the combined themes, all mothers stated that the themes reflected their experience appropriately. A few minor word changes to provide more specificity were made by the mothers and added to the themes.

To support transferability, detailed descriptions were created of the environment in which the data were collected, the individuals being studied, and the context of the study. Readers will be able to determine whether these findings are applicable to other environments. To support dependability, an audit trail was maintained listing every methodological decision made while collecting and analyzing data. This includes coding books and analytical memos. The confirmability of the study was increased through the use of reflective journals, the bracketed practice discussed in Section 2.9, and a peer reviewer who was an independent qualitative researcher and also a senior nursing professor with knowledge of qualitative methods. Feedback from the peer reviewer assisted in challenging interpretations of themes, refining label names of sub-themes, and ensuring that there was no overlap between thematic boundaries. Reporting of this study followed the Consolidated Criteria for Reporting Qualitative Research (COREQ) 32-item checklist (Supplementary File S2).

### 2.8. Ethical Considerations

The study was approved by the Health Cluster Institutional Review Board (IRB) in Hail (approval number H-08-L-187) on 11 July 2026 before any data were collected. The study was implemented in compliance with the Declaration of Helsinki. An Arabic-language Information Sheet outlining the aims and methods of the study, along with the risks and benefits of participation and the participant’s rights, was given to each participant. Written informed consent was completed before the interviews began. Written permission for audio recording was also obtained. Participation was voluntary. Participants could refuse to answer any question or withdraw at any stage without fear of consequence. Confidentiality was ensured through the use of pseudonyms in the transcriptions, removal of all identifiable information, and safe, encrypted storage of recorded materials and field notes. These materials can be accessed only by members of the research group. No financial inducements were provided. No participants withdrew from the study after providing informed consent, and no adverse psychological or emotional distress events occurred during or following the interviews.

### 2.9. Rigor and Reflexivity Statement

The researchers are all practicing nurses with prior experience promoting breastfeeding clinically. The team recognized that this background could shape both how participants answered and how data were interpreted. For this reason, bracketing was practiced as an ongoing effort rather than a one-time step. The researchers kept a reflexive journal throughout the study. They met regularly with a qualitative researcher as they analyzed the data, for the purpose of challenging developing interpretations. The aim of these practices was faithful description rather than imposed interpretation, which is central to descriptive phenomenology [18,20].

## 3. Results

A total of 13 working mothers, aged 27 to 32 years (average 29.5), completed the study. Participants were recruited through both purposive and snowball sampling, including mothers attending maternal and child health services at public primary care clinics in Hail City, and they represented employment sectors including government, education, healthcare, and private industry. The infants’ ages ranged from three to ten months.

Analysis of the verified English translations (original Arabic transcripts and audio recordings were consulted for clarification when needed) using Colaizzi’s seven-step process yielded five themes consisting of multiple subthemes. These five themes represent the essential characteristics of the transitional process experienced by breastfeeding working mothers returning to work. Quotations below are translated from Arabic. Culturally specific expressions were kept where possible, and the translation was checked by a second bilingual researcher who verified linguistic fidelity and cross-language conceptual equivalence against the original Arabic audio recordings. Quotations are reproduced verbatim from the verified English translations; metaphorical or emotionally expressive phrasing reflects participants’ own words in Arabic and was retained rather than normalized. Where participants’ accounts differed from the main pattern, these differences are described within the relevant themes: one mother whose workplace provided a dedicated, private lactation room (Theme 2), and one participant whose supervisor treated the nursing break as a customary entitlement rather than a favor (Theme 3).

Table 1 presents the demographic characteristics of the participants. There are six mothers who are primiparous (46.2%) and seven are multiparous (53.8%). Maternity leave duration is as reported by participants and reflects the leave they actually took, which could be shorter or longer than the statutory entitlement. Civil Service employees are entitled to 60 days of fully paid maternity leave, while private-sector employees under the Labor Law are entitled to 12 weeks (84 days), increased from 10 weeks with effect from 18 February 2025. Several participants reported 70-day leaves, which exceed the statutory entitlement in their sector. These most likely reflect statutory leave combined with additional employer-granted or accrued leave. Breastfeeding status reflects feeding practices at the time of the qualitative interview.

All 13 participants were continuing to provide breast milk at the time of interview, whether exclusively or in combination with formula or solids. Throughout the Results section, we report the approximate number of participants contributing to each pattern. These counts are provided for transparency regarding the consistency of each pattern within this small sample; they are descriptive of this sample only and are not prevalence estimates.

Two variations within the sample are worth reporting for transparency. First, feeding status at interview was associated with time since return: all six mothers interviewed within 2.5 months of returning to work (P1, P4, P7, P9, P11, P13) were exclusively providing breast milk and expressing milk during work hours, whereas among the seven mothers interviewed three months or more after return, six had introduced formula or solids, and one (P5) remained exclusive. Second, the two workplace experiences that diverged from the dominant pattern (P1’s dedicated lactation room and P13’s unproblematic use of the nursing break) both occurred in the government sector, and no comparable positive account came from the three private-sector participants. Leave duration followed a similar though imperfect pattern: five of the six mothers with 70-day leave were exclusively breastfeeding at interview, compared with two of the seven with 60-day leave. With 13 participants, and with sector, employer, and leave duration intertwined, these patterns are descriptive of this sample only; no causal or generalizable interpretation is drawn from them.

Table 2 shows examples of the progression from participants’ significant statements, to formulated meanings, to the themes they informed.

### 3.1. Theme 1: Return: Anticipation, Preparation, and Quiet Resolve

During this phase, mothers looked ahead to balancing the two major responsibilities in their lives, work and breastfeeding, before returning to work. Mothers developed intentional, sometimes individualized plans to meet both responsibilities. This time frame also included mothers adjusting their personal breastfeeding goals once they knew how many days remained before their return to work.

#### 3.1.1. Subtheme 1.1: Planning During Maternity Leave

Mothers used both practical and anticipatory preparation strategies during maternity leave to support continued lactation after returning to work. These included creating a stockpile of frozen expressed milk, practicing with a breast pump before returning, helping the infant get used to being fed from a bottle, and arranging for the care of their child. All but one participant reported building an inventory of expressed milk before returning to work. They also thought through pumping schedules based on their expected working hours. Planning was an individual undertaking. None of the mothers had received guidance on return-to-work breastfeeding preparation from a healthcare provider.

“*I began to freeze milk in the second month of my maternity leave. My freezer looked as though it belonged in a pharmacy. Every time I opened my freezer, I would count how many bags were there and tell myself, if I can get ready enough, then work will never be able to beat me.*”

(P2)

“*The idea that no one told me what to do is funny. At night when the baby was sleeping I watched videos on breastfeeding and pumping, and I used my breast pump until it stopped hurting.*”

(P6)

#### 3.1.2. Subtheme 1.2: Lowering Expectations, from Exclusive to Partial Breastfeeding

Mothers gradually revised their feeding goals from exclusive breastfeeding to partial or mixed breastfeeding in the weeks before their first working day. They made this change as they realistically assessed their workplace conditions. Participants viewed this decision as an early concession. They saw it as a response to expected time limits and limited facilities, rather than as a free choice.

“*And before I left, I said to myself, exclusive breastfeeding is over. All I can wish for at this point is to be able to give her at least one feeding per day. In my heart, I didn’t want to accept this. I didn’t want to fail completely.*”

(P8)

### 3.2. Theme 2: The Workplace as an Unaccommodating Space

This theme illustrates the physical and logistical barriers mothers faced when they returned to work. These included lack of a lactation room at the workplace for expressing and storing breast milk, no reliable refrigeration for milk storage, and lack of protected time. These barriers led mothers to find makeshift, unhygienic, or humiliating arrangements to express and store breast milk at work.

#### 3.2.1. Subtheme 2.1: Nowhere to Express

Nearly all mothers (12 out of 13) reported not having access to a private space for expressing. These spaces included washrooms, storerooms, prayer rooms and other communal areas. The lack of a lockable, private space for milk expression was reported as a daily source of stress and was cited by some participants as a reason for stopping milk expression. P1, an administrative clerk in the government sector, was the only participant (out of 13) to report that there was a dedicated lactation room available in her workplace. The room had a locked door and there was a refrigerator near her workstation. She did not have to hide expressing from her co-workers or supervisors and expressed on schedule. P1 attributed her decision to continue breastfeeding to this support at work.

“*They looked at me like I had asked to do something weird when I requested a space to pump.*”

(P6)

“*I pumped in the bathroom and threw the milk away since it wasn’t safe enough to store in that bathroom. To throw out your own milk is just too much. That’s why it broke my heart.*”

(P4)

#### 3.2.2. Subtheme 2.2: Time Pressure

The mothers explained that they often could not express milk during short breaks because of high student traffic or work demands. At other times they expressed at inappropriate times, such as after meals, because no private location was available. They reported frequently missing scheduled expressing times. They also described severe engorgement, which caused leakage through clothing, and decreased milk production.

“*It was hard enough to find time to eat, let alone time to pump.*”

(P9)

“*By lunchtime my breasts had become very sore and swollen, and I started wearing darker clothing so that no one would notice the leaking.*”

(P12)

#### 3.2.3. Subtheme 2.3: Milk Storage, Hygiene, and Improvisation

Mothers described the difficulty of storing expressed milk safely at work without a dedicated refrigerator. Some used shared refrigerators secretly. Others used coolers and worried about possible contamination of their milk. Mothers also reported the hidden costs of delaying expression, including engorgement and, in some cases, mastitis. They linked these problems to the physical discomfort caused by the lack of support at work.

“*I think three times, I carried my expressed breast milk in an insulated cooler. That was a secret each day… it became apparent that there could be a variety of outcomes on a given day.*”

(P11)

Later in her return, once she had identified a storage option, the same mother (P4) described an improvised solution: “*My breasts felt full and heavy. My friend told me to express and store the milk, so I placed the expressed milk in a small refrigerator at my workplace.*” (P4)

### 3.3. Theme 3: Between the Law on Paper and the Law in Practice: The Nursing-Break Entitlement

Formal rights exist under Article 154 of the Saudi Labor Law, which allows nursing employees a breastfeeding break of up to one hour per day [9]. In the participants’ accounts, this right was largely unknown, and access to it appeared to depend heavily on the goodwill of supervisors. This theme captures the gap between these formal protections and mothers’ lived experiences.

#### 3.3.1. Subtheme 3.1: A Right Nobody Knows

Participants lacked knowledge of the legal nursing-break entitlement before the study. Legal knowledge traveled informally between mothers rather than through employers. Only two participants had heard of the provision before returning to work, and none had been informed of it by their employer. One further participant used the nursing break as an established workplace custom rather than as a known legal entitlement (Subtheme 3.2).

“*Nobody ever told me about the nursing hour. I found out about it when one of my coworkers got embarrassed for even requesting it. So after that I chose to remain silent, in order to protect myself from being humiliated.*”

(P5)

“*A co-worker of mine told me about it. I was only informed when she said that when she had a baby before, she was also informed by our coworker.*”

(P8)

#### 3.3.2. Subtheme 3.2: Supervisor Discretion and Informal Negotiation

Mothers who continued to use the nursing break described instances in which their supervisors treated it as a favor that could be granted or withheld. Mothers who stopped requesting or using the break described supervisors as unresponsive to their requests; some also reported a subsequent decline in milk supply or increased difficulty maintaining milk expression. Access to the nursing break therefore appeared to depend on the supervisor rather than on a consistently implemented workplace entitlement. However, this was not the case for all mothers. P13’s manager treated the nursing break as a customary entitlement, and P13 did not need to request permission; she simply gave informal notice that she was leaving for her break. In this case, Article 154 operated substantially as intended. Overall, participants’ accounts suggest that supervisors’ interpretation and implementation of the entitlement shaped whether mothers could use it in practice.

“*The law requires one hour, but my manager said we are understaffed, so she felt I had taken advantage by taking an extra hour off work. I then stopped requesting the hour. It wasn’t long until my milk ceased to flow also, as if my body realized there was now no place for the milk in my system either.*”

(P9)

#### 3.3.3. Subtheme 3.3: Concealment: Hiding Pumping to Avoid Judgment

Mothers made deliberate efforts to keep pumping invisible. Their goal was to protect their professional image and avoid being labeled as less committed employees. Mothers timed sessions to avoid colleagues, gave false reasons for absences from their desks, and avoided disclosure to male coworkers.

“*I was never comfortable enough with my male supervisor to explain to him that I need to leave my desk.*”

(P6)

“*Sometimes I would ask permission and tell them I had a doctor’s visit. Other times I wouldn’t mention it. It felt like being a mom at work was something I should have apologized for.*”

(P10)

### 3.4. Theme 4: Straddling Two Worlds: Identity, Guilt, and Emotional Labor

This theme describes the internal experience of the transition. It covers the renegotiation of what it means to be a good mother and a good employee at the same time. It also covers the emotional labor of managing guilt, anxiety, and divided loyalty in both roles.

#### 3.4.1. Subtheme 4.1: A Divided Self: Renegotiating Maternal and Professional Identity

Mothers described identity work as they tried to remain fully present in two demanding roles. At the same time, the workplace seemed to expect the pre-maternal employee and offered no legitimate category for the lactating maternal body.

“*Work has always been about being the old me, the organized, productive person. But as a mother my body is no longer only that woman. In meetings when I feel my milk let down, it is like two people fighting to occupy the same seat.*”

(P12)

“*I absolutely adore both my daughter and my profession. However, no one told me how much I would miss my daughter at 10 am, and the tremendous guilt I would feel from missing her in order to do my job.*”

(P13)

#### 3.4.2. Subtheme 4.2: The Emotional Toll: Guilt, Anxiety, and Exhaustion

Mothers described persistent difficult emotions during the transition. These included guilt toward the infant during hours of separation, guilt toward employers for time spent pumping, anxiety about milk supply, and exhaustion from night feeds followed by full working days. Some mothers cried during interviews when recounting the first weeks back.

“*When I left my baby I felt like a bad mother, and when I took time to pump I felt like a bad employee. I felt I was failing at both, in my own head, every single day.*”

(P1)

“*I would be driving to work asking myself why I need to endure this. I cried, but I must be strong.*”

(P13)

### 3.5. Theme 5: Circles of Support: Family, Peers, and Inner Resolve

This theme illustrates how mothers were able to continue breastfeeding despite the obstacles they encountered. It includes a mother’s personal determination and confidence in her ability to breastfeed, as well as emotional and practical help from family and female colleagues and supervisors.

#### 3.5.1. Subtheme 5.1: Family as the Backbone of Continued Breastfeeding

Practical help came from husbands, mothers, and mothers-in-law. This included caring for the infant during working hours, bringing the infant to the workplace or nearby for direct feeds, preparing expressed-milk feeds, and offering sustained emotional encouragement. Many participants described family support as the most important facilitator of their continued breastfeeding.

“*Every single day of the two months that she stayed at the health center with me, my husband came to take our baby to the health center so I could pump. If he hadn’t taken care of this for me, my milk would have dried out by week one.*”

(P9)

“*I must be thankful, as my mother is always by my side. You know, especially when I come home from work, she helps me prepare before I breastfeed my child.*”

(P7)

#### 3.5.2. Subtheme 5.2: Coworker Solidarity and Peer Normalization

Supportive colleagues covered tasks during pumping breaks, shared information and equipment, and normalized breastfeeding at work. Mothers experienced such support as rare and precious. Where such support was absent, and where mothers faced mockery or complaints of favoritism, they described greater difficulty continuing to express milk at work.

“*One of the senior nurses would guard my door while I pumped and tell everyone I was in meetings. She had gone through it all before. I will always be thankful for what she did for me.*”

(P3)

#### 3.5.3. Subtheme 5.3: Determination and Self-Efficacy: The Inner Resource

Mothers held a personal conviction that breastfeeding mattered and felt confident in their capacity to sustain it against obstacles. Participants described previous positive breastfeeding experience and strong feeding knowledge as important sources of confidence and determination in their efforts to continue breastfeeding.

“*I decided before I went back that I would breastfeed for one year, whatever it takes. The bathroom, the cooler, the looks, all of it.*”

(P8)

### 3.6. Fundamental Structure of the Experience

**Figure 1:**
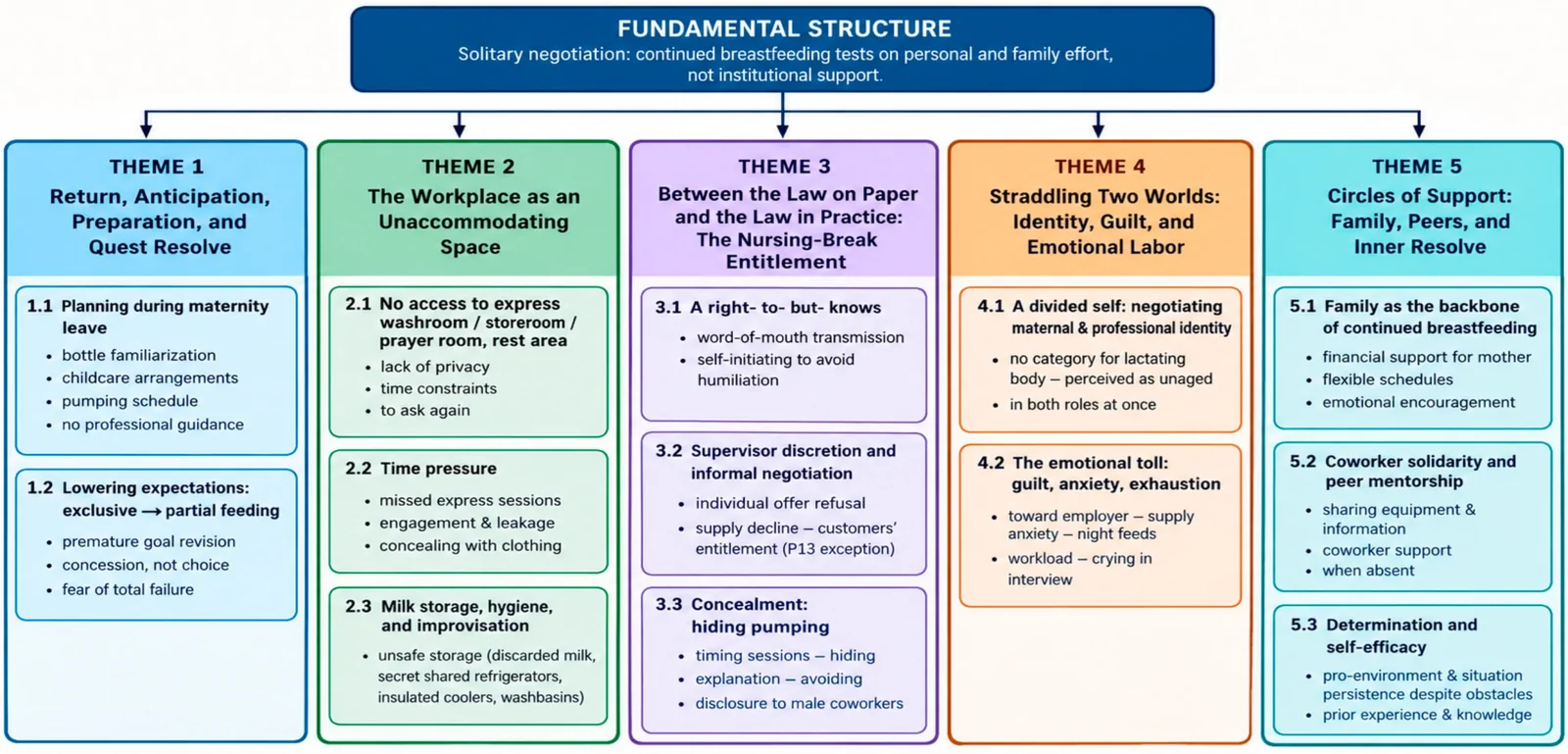
Coding tree showing the hierarchical structure from descriptive codes to subthemes and themes, synthesized into the fundamental structure of the experience.

Bringing the five themes together, the research team described the core of the experience as solitary negotiation. The mother returns to work with a legally recognized right she often does not know she has. She enters a workplace that appears not to have been designed with her lactating body in mind. She negotiates pumping arrangements largely alone, and she continues breastfeeding largely because of the efforts of her family or her own internal motivation. This structure is the team’s synthesis of the five themes. It is grounded in the participants’ accounts and was checked with them during member checking, rather than being any single participant’s own words. The complete coding tree, from descriptive codes through subthemes and themes to the fundamental structure, is presented in Figure 1.

## 4. Discussion

The present research examined the lived experiences of employed mothers living in Hail City, Kingdom of Saudi Arabia, who were returning to employment from maternity leave while continuing to breastfeed. Because all mothers in the study were still providing breast milk at the time of the interview, we are unable to comment on the experiences of mothers who ceased providing breast milk before the interview. Rather, our discussion is limited to the experiences of mothers who continued at least some breastfeeding after returning to work. The five themes described in this study show how these women negotiated a transition that was, in their accounts, largely solitary. They prepared for the return individually during maternity leave. They returned to workplaces with little or no access to lactation rooms or time to express milk at work. They had rights under the law that many were unaware of. They carried emotional distress and an internally divided identity. Each theme is discussed below in relation to previous research. As a descriptive phenomenological study, the analysis itself was carried out without drawing on theory. The theories mentioned below were used after the themes had emerged, as lenses for interpreting the findings in light of the wider literature.

### 4.1. Return: Anticipation, Preparation, and Quiet Resolve

That mothers began preparing during maternity leave, and lowered their feeding goals before their first working day, is consistent with quantitative evidence that the timing of return to employment and the length of maternity leave powerfully shape breastfeeding trajectories. In a survival analysis of nationally representative Scottish data, Skafida found that employment was associated with earlier breastfeeding cessation and that longer maternity leave was protective, operating through delayed return [25]. Similarly, scholars found that employment characteristics in the United States were associated with both breastfeeding initiation and duration, with longer maternity leave supporting continued breastfeeding [26]. The finding that mothers in Hail pre-emptively revised their goals from exclusive to partial breastfeeding is also consistent with previous qualitative evidence that women’s experience, knowledge, and expectations shape how they plan for and persevere with breastfeeding after returning to paid employment [27]. Crucially, the Hail mothers planned alone. None had received return-to-work breastfeeding preparation from a healthcare provider. This represents a missed opportunity for anticipatory support during routine postnatal contacts, the very window in which mothers have their most frequent contact with nurses and midwives.

### 4.2. The Workplace as an Unaccommodating Space

The near-absence of lactation rooms, protected time, and safe milk storage in this sample echoes the most consistent findings of the international literature. In their systematic review of studies of working women in the United States, researchers identified lack of time and lack of suitable space as the dominant barriers to breastfeeding at work [28]. The quantitative stakes of these barriers were demonstrated by scholars who found that only 40% of employed U.S. mothers had access to both adequate break time and a private space to express milk. Women with both accommodations had more than twice the odds of exclusive breastfeeding at six months [29]. The present findings also align with this Saudi quantitative evidence; what the qualitative accounts add is a description of how mothers experience, negotiate, and improvise around that absence in everyday working life. Across these studies, lactation rooms appear to be absent in the large majority of surveyed Saudi workplaces, although the present qualitative study cannot establish national prevalence. Most sobering is the comparison with researchers who found that most of the nine participating hospital employees had ceased breastfeeding before their infants were six months old [13]. In the absence of institutional accommodations, family-driven workarounds like midday infant visits seemed to support breastfeeding among the mothers in our sample, all of whom were still providing breast milk at the time of the interview. In a South African government workplace, researchers similarly documented how the absence of workplace facilities undermined mothers’ efforts to continue breastfeeding [30].

### 4.3. Between the Law on Paper and the Law in Practice: The Nursing-Break Entitlement

That participants either did not know of, or could not use, the Article 154 entitlement mirrors a gap between breastfeeding legislation and practice documented worldwide. It should be noted that, since participants worked in different sectors, this study did not ascertain whether Article 154 was applicable to them in exactly the same way across public-sector, educational, and private employment. This is relevant because some of the practices described by participants may reflect limited knowledge of their legal entitlements or practices that fall short of those entitlements, rather than actual legal non-compliance. Such a gap between law and practice has been documented extensively and is by no means specific to Saudi Arabia. For example, although a law passed in 2010 requires employers to provide reasonable breaks for working mothers in the U.S., many working mothers do not receive these breaks, particularly in small businesses [6]. When researchers synthesized data from qualitative studies across multiple countries, they concluded that legislative action alone is insufficient. Communication between women and their managers, and education within the workplace, are also needed [27]. Two theoretical frameworks help interpret why supervisor discretion appeared to prevail over policy in the participants’ accounts. First, Perceived Organizational Support Theory suggests that employees react to the discretionary treatment they receive from an organization as if it reflects whether the organization values them [31]. When a supervisor gives a nursing employee an hour to breastfeed or express milk, she may read this as evidence that she is valued. When the break is denied, she may read it as the opposite. Second, research on breastfeeding in organizations has empirically shown that social support from coworkers and supervisors greatly surpasses other forms of organizational support. Support from colleagues and supervisors has been identified as necessary for continued exclusive breastfeeding [32]. However, as demonstrated in a number of studies [7], these forms of support are often absent. Returning mothers therefore rely primarily on family and partners, rather than their employer, for most of their support during the process of returning to work while breastfeeding. For policymakers, these findings suggest that enforcing the legislation, raising awareness, and training supervisors are directions worth pursuing and evaluating.

### 4.4. Straddling Two Worlds: Identity, Guilt, and Emotional Labor

The guilt, anxiety, and divided identity described by participants can be interpreted through work-family conflict theory, which proposes that conflicts between work and family roles arise when involvement in one role makes it more difficult to participate in the other [33]. Mothers described two different selves, like two women fighting inside one chair. They felt simultaneous guilt toward both their infant and their employer. In the participants’ accounts, this conflict was intensified where employers made little room for the overlap between the two roles. The emotional content of the finding mirrors the international qualitative evidence. Researchers identified maternal emotional difficulties, including guilt, stress, anxiety, and embarrassment, as a recurring synthesized finding across countries, together with the concealment of breastfeeding at work [27]. In their qualitative study of breastfeeding mothers returning to work in Ireland, researchers similarly described workplaces that treated feeding as incompatible with professional identity, and mothers who felt under pressure as their return date approached [34]. The Hail mothers’ concealment strategies, and their sense that motherhood at work was something to apologize for, therefore show clear similarities with this internationally documented pattern. At the same time, the specific role of extended family in Hail may give these experiences a locally distinctive form, an area that future cross-cultural research could examine.

### 4.5. Circles of Support: Family, Peers, and Inner Resolve

Participants’ accounts of how family, coworkers, and personal determination helped them sustain breastfeeding align with the earlier synthesis. Scholars identified support from partners, family members, colleagues, and employers as the key facilitator of breastfeeding continuation after return to employment across diverse countries [27]. Researchers also showed that where institutional support is absent, mothers’ persistence depends on precisely such informal circles [30]. On maternal determination, the finding aligns with breastfeeding self-efficacy theory, which holds that a mother’s confidence in her ability to breastfeed is a modifiable determinant of breastfeeding outcomes [35]. This was operationalized in the Breastfeeding Self-Efficacy Scale [35]. Participants often linked a firm pre-return commitment to breastfeeding with their efforts to continue despite workplace obstacles. In Saudi Arabia, mothers who worked were less likely to initiate and had shorter duration of breastfeeding [10]. Exclusive breastfeeding rates in infants under six months were 8.86 percentage points higher in countries that guarantee paid breastfeeding breaks for at least six months [4]. Vision 2030 anticipates a substantial rise in the proportion of women in Saudi Arabia’s workforce. In order to meet the World Health Organization’s global nutrition objectives [1], Saudi Arabia can establish national standards for lactation support in the workplace. To transform this support ecology into public health policy would require shifting from circumstance-based support (e.g., when a husband is available to make the lunchtime trip, or a coworker to stand guard by the door) to enforceable lactation protections in the workplace. Future research is needed to explore whether self-efficacy serves as a buffer to unsupportive work environments and further examine the importance of social support for breastfeeding in the workplace.

### 4.6. Study Implications

The implications of the results of this study are related to nursing practice and lactation support, organizational practices, enforcement of Article 154 of the Saudi Labor Law, and conducting future studies. Healthcare providers during postnatal contacts should include breastfeeding support interventions such as counseling on methods of storing expressed breast milk (refrigeration/freezing), pumping schedule, and education about the rights of breastfeeding women before returning to work. In addition, provision of lactation consultant support during the first weeks after returning to work is recommended.

At the organizational level, having a designated area for breastfeeding and expressing milk (with a refrigerator, when possible) and considering expressing time in the workload are recommended. The study results also emphasize the importance of providing information and training programs for supervisors/representatives of employers to consider providing support to pumping/breastfeeding employees as their responsibility rather than a favor. Coworkers can play a vital role by supporting their peers.

The results of this study can be used to enforce and increase awareness of Article 154 of the Saudi Labor Law through conducting education/campaigns targeting employees and employers, in addition to providing the facilities mentioned in the article. More studies are needed to assess the effectiveness of the interventions at the workplace level (having facilities, providing training for supervisors, etc.) and to compare the experiences of working mothers in different settings (public/private sectors) and different areas in the Kingdom of Saudi Arabia.

### 4.7. Study Limitations

The study was conducted in only one mid-sized city, thus limiting transferability to other parts of Saudi Arabia that might have a different employment makeup. The results reflect the situation in Hail City, not necessarily what might be happening in workplaces throughout the country. The use of purposive and snowball sampling may also have introduced selection bias. Mothers who were successfully continuing breastfeeding, or who had strong family support, may have been more visible in referral networks than mothers who weaned immediately upon returning to work. Moreover, all 13 participants were still breastfeeding at the time of interview. Therefore, the findings primarily reflect the experiences of mothers who maintained at least some breastfeeding after returning to work. They do not capture the experiences of mothers who stopped breastfeeding before the interview and cannot identify characteristics that distinguish continuation from cessation. Third, all interviewers were nurses with breastfeeding expertise. This may have encouraged socially desirable responses, even though efforts were made to put participants at ease. Recruitment through professional and clinical networks may also have shaped who felt comfortable participating. Fourth, interviews were conducted in Arabic and are reported in English. Although the translation was verified by a second bilingual researcher through independent cross-checks against the original Arabic audio recordings and selected back-translation checks, some nuance of tone and culturally specific expression may have been altered. Fifth, limiting eligibility to infants under one year means the experiences of mothers of older infants, multiparous mothers returning after longer gaps, and mothers in precarious or informal employment are not represented. Sixth, the demographic and breastfeeding trajectory data collected were limited, so the study cannot establish causal or generalizable relationships between these variables and breastfeeding status. Finally, the cross-sectional, single-interview design captured the experience at one point in time. The transition unfolds over months, and a longitudinal design could have traced how negotiations with employers evolved over time.

## 5. Conclusions

The purpose of this study was to describe, using Husserlian descriptive phenomenology and Colaizzi’s method, the lived experiences of employed mothers in Hail City who returned to employment while continuing to breastfeed. Within this sample, the experience was characterized by solitary negotiation. Participants prepared individually during maternity leave and returned to workplaces that generally offered limited support for lactating employees. Many were unaware of the nursing-break entitlement, and their accounts described concealing milk expression and feelings of guilt to preserve a competent professional image. Among these mothers, all of whom were still providing breast milk at interview, maintaining breastfeeding was supported largely by personal commitment and informal practical help from family and coworkers. These findings reflect the experiences of 13 mothers in one city who were able to maintain at least some breastfeeding after returning to work. They do not represent mothers who ceased breastfeeding before interview and should not be read as evidence of conditions nationwide. Within these limits, the findings point to directions that merit attention as female workforce participation grows under Vision 2030: implementing Article 154 in everyday practice, providing functional workplace lactation spaces, training supervisors to support nursing mothers, and establishing clinical support systems before mothers return to work. Each of these directions should be evaluated in future research rather than assumed to work.

## Figures and Tables

**Table 1:** Demographic Characteristics of the Participants (*n* = 13).

Participant	Age (yrs)	Occupation	Employment Sector	Parity	Infant Age (Months)	Maternity Leave Duration	Time Since Returning to Work	Breastfeeding Status at Interview
P1	27	Administrative clerk	Government	Primiparous (1)	4	70 days	1.5 months	Exclusive (expressing at work)
P2	29	Schoolteacher	Education	Multiparous (2)	6	60 days	4 months	Mixed (breast milk + formula)
P3	32	Registered nurse	Healthcare	Multiparous (3)	8	70 days	5.5 months	Mixed (breast milk + formula)
P4	28	Bank teller	Private	Primiparous (1)	3	60 days	1 month	Exclusive (expressing at work)
P5	31	University administrator	Education	Multiparous (2)	7	70 days	4 months	Exclusive (expressing at work)
P6	30	School administrator	Education	Multiparous (2)	5	60 days	3 months	Mixed (breast milk + solids)
P7	29	Registered nurse	Healthcare	Primiparous (1)	4	70 days	1.5 months	Exclusive (expressing at work)
P8	32	Retail supervisor	Private	Multiparous (3)	10	60 days	8 months	Mixed (breast milk + solids)
P9	27	Clinic receptionist	Healthcare	Primiparous (1)	3.5	60 days	1.5 months	Exclusive (expressing at work)
P10	30	Accountant	Private	Multiparous (2)	6	60 days	4 months	Mixed (breast milk + formula)
P11	28	Laboratory technician	Healthcare	Primiparous (1)	5	70 days	2.5 months	Exclusive (expressing at work)
P12	31	Schoolteacher	Education	Multiparous (3)	9	60 days	7 months	Mixed (breast milk + solids)
P13	29	Social worker	Government	Primiparous (1)	4.5	70 days	2 months	Exclusive (expressing at work)

**Table 2:** Examples of the Progression from Significant Statements to Formulated Meanings and Themes.

Theme	Significant Statement (Participant’s Words)	Formulated Meaning	Theme/Subtheme It Informed
1. Return: Anticipation, Preparation, and Quiet Resolve	“I began to freeze milk in the second month of my maternity leave… Every time I opened my freezer, I would count how many bags were there and tell myself, if I can get ready enough, then work will never be able to beat me.” (P2)	Preparation for return to work is undertaken alone during maternity leave and experienced as a private battle to build enough milk reserves to withstand an unsupportive workplace.	Theme 1, Subtheme 1.1: Planning during maternity leave
2. The Workplace as an Unaccommodating Space	“They looked at me like I had asked to do something weird when I requested a space to pump.” (P6)	Requesting a private space to express milk was experienced as an abnormal or illegitimate demand; mothers described hesitating to ask again afterward.	Theme 2, Subtheme 2.1: Nowhere to express
3. Between the Law on Paper and the Law in Practice	“The law requires one hour, but my manager said we are understaffed, so she felt I had taken advantage by taking an extra hour off work. I then stopped requesting the hour. It wasn’t long until my milk ceased to flow also…” (P9)	The statutory nursing break was experienced as existing on paper only, its use hinging on the supervisor’s personal judgment; mothers described abandoning the break when it was framed as abuse rather than entitlement, followed by a decline in milk supply.	Theme 3, Subtheme 3.2: Supervisor response and informal negotiation
4. Straddling Two Worlds: Identity, Guilt, and Emotional Labor	“When I left my baby I felt like a bad mother, and when I took time to pump I felt like a bad employee. I felt I was failing at both, in my own head, every single day.” (P1)	Guilt toward the infant during separation and guilt toward the employer for time spent pumping were described as coexisting and renewing daily, with the mother experiencing herself as failing in both roles at once.	Theme 4, Subtheme 4.2: The emotional toll: guilt, anxiety, and exhaustion
5. Circles of Support: Family, Peers, and Inner Resolve	“One of the senior nurses would guard my door while I pumped and tell everyone I was in meetings. She had gone through it all before. I will always be thankful for what she did for me.” (P3)	Informal protection provided by an experienced female colleague makes expressing milk possible at work; such coworker solidarity is experienced as rare and precious.	Theme 5, Subtheme 5.2: Coworker solidarity and peer normalization

## Data Availability

The data presented in this study are available on request from the corresponding author.

## Supplementary Materials

The following supporting information can be downloaded at https://www.mdpi.com/article/10.3390/healthcare14213766/s1, File S1. Semi-Structured Interview Guide. File S2. COREQ Checklist [36].
