# Supplementary material for: The Lived Experience of Employed Mothers Transitioning Back to the Workplace While Breastfeeding
Source: Healthcare (Basel). 2026 Nov 4;14(21):3766. doi: 10.3390/healthcare14213766 (PMC13763014; doi:10.3390/healthcare14213766)
Supplement: Supplementary file 1 [file healthcare-14-03766-s001.zip › healthcare-4635926 - Supplementary File S1 - Interview_Guide.pdf]

Supplementary File S1. Semi-Structured Interview Guide

(English Version)

The Lived Experience of Employed Mothers Transitioning Back to the Workplace While Breastfeeding

Version 2.0 (post-pilot), 21 July 2026. This English version is the verified translation of the guide used to conduct interviews in Arabic. The guide was pilot-tested with one eligible mother; Version 2.0 revisions clarified technical language, reordered more psychologically sensitive questions to later in the interview, and added follow-up probes on time limitations during working hours. Pilot data were excluded from analysis.

Pre-interview script

- Introduce self; confirm informed consent and written permission for audio recording.
- Reconfirm voluntarism: participation is strictly voluntary; declining to answer any question or withdrawing will not affect medical care or services for the participant or her infant.
- Reconfirm privacy of the setting chosen by the participant; estimated duration 30–50 minutes.
- Explain that there are no right or wrong answers and that she may pause or stop at any time.

Opening (rapport)

Tell me a little about your baby and how things have been going since you returned to work.

Grand tour question

"Describe your experience of returning to work while continuing to provide breast milk to your infant."

Topic areas and probe questions (use as needed)

1. Anticipatory preparation for the end of maternity leave

- How did you prepare during maternity leave for continuing breastfeeding after returning to work? (freezer stockpile, pump practice, bottle introduction, childcare arrangements)
- What information or guidance did you receive from healthcare providers before returning? What did you wish you had been told?
- How did your feeding goals change as your return date approached?

2. First days back and adjustment

- Walk me through a typical working day since you returned — when and where do you express milk?
- What has been easiest and hardest about combining work and breastfeeding so far?

- Follow-up (pilot addition): Tell me about time limitations during working hours and how they affect expressing.

3. The nursing hour (Article 154) and lactation facilities
- Had you heard of the one-hour daily breastfeeding break before returning? How did you learn about it?
- Tell me about any designated space for expressing or storing milk at your workplace.
- What happened when you asked to use the break or the space? (probe: supervisor response, conditions, whether use continued)

4. Interactions with supervisors and colleagues
- How have your supervisor(s) responded to your need to express milk at work?
- How have colleagues responded? Have you received help, or felt judged? (probe: concealment strategies; comfort discussing with male vs. female colleagues)

5. Sources of support
- Who has helped you keep breastfeeding since you returned — family, husband, coworkers, employer, healthcare providers?
- What kind of help has mattered most? What support is missing?

6. Emotional and physical experience
- How has this experience affected you emotionally — guilt, anxiety, exhaustion?
- How has it affected you physically (engorgement, milk supply, mastitis, fatigue)?

7. Meaning as a mother
- What does continuing to breastfeed while working mean to you as a mother?
- Has returning to work changed how you see yourself as a mother or as an employee?

Closing
- Is there anything important about your experience that I have not asked about?
- Thank the participant; remind her of confidentiality arrangements; administer the short demographic questionnaire.
