# Supplementary material for: The Lived Experience of Employed Mothers Transitioning Back to the Workplace While Breastfeeding
Source: Healthcare (Basel). 2026 Nov 4;14(21):3766. doi: 10.3390/healthcare14213766 (PMC13763014; doi:10.3390/healthcare14213766)
Supplement: Supplementary file 1 [file healthcare-14-03766-s001.zip › healthcare-4635926 - Supplementary File S2 - COREQ Checklist.pdf]

Supplementary File S2. COREQ (COnsolidated criteria for REporting Qualitative research) Checklist

Manuscript: Shanmugam et al. “The Lived Experience of Employed Mothers Transitioning Back to the Workplace While Breastfeeding” — revised version, 36 pages.

Topic	Item No.	Guide Questions/Description	Reported on Page No.
**DOMAIN 1: RESEARCH TEAM AND REFLEXIVITY**			
**Personal characteristics**			
Interviewer/facilitator	1	Which author/s conducted the interview or focus group?	5
Credentials	2	What were the researcher's credentials? E.g. PhD, MD	5
Occupation	3	What was their occupation at the time of the study?	5
Gender	4	Was the researcher male or female?	5
Experience and training	5	What experience or training did the researcher have?	5
**Relationship with participants**			
Relationship established	6	Was a relationship established prior to study commencement?	5
Participant knowledge of the interviewer	7	What did the participants know about the researcher? e.g. personal goals, reasons for doing the research	5
Interviewer characteristics	8	What characteristics were reported about the interviewer/facilitator? e.g. bias, assumptions, reasons and interests in the research topic	5
**DOMAIN 2: STUDY DESIGN**			
**Theoretical framework**			
Methodological orientation and theory	9	What was the methodological orientation and theory? e.g. grounded theory, discourse analysis, ethnography, phenomenology, content analysis	3
**Participant selection**			
Sampling	10	How were participants selected? e.g. purposive, convenience, consecutive,	4

		snowball	
Method of approach	11	How were participants approached? e.g. face-to-face, telephone, mail, email	4
Sample size	12	How many participants were in the study?	4
Non-participation	13	How many people refused to participate or dropped out? Reasons?	5
**Setting**			
Setting of data collection	14	Where was the data collected? e.g. home, clinic, workplace	4
Presence of non-participants	15	Was anyone else present besides the participants and researchers?	8
Description of sample	16	What are the important characteristics of the sample? e.g. demographic data, date	8
**Data collection**			
Interview guide	17	Were questions, prompts, guides provided by the authors? Was it pilot tested?	5
Repeat interviews	18	Were repeat interviews carried out? If yes, how many?	Not conducted — single interview per participant; member checking instead (item 28)
Audio/visual recording	19	Did the research use audio or visual recording to collect the data?	6
Field notes	20	Were field notes made during and/or after the interview or focus group?	6
Duration	21	What was the duration of the interviews or focus group?	6
Data saturation	22	Was data saturation discussed?	4
Transcripts returned	23	Were transcripts returned to participants for comment and/or correction?	Not returned verbatim — plain-language synthesized summary returned for member checking instead (11)
**DOMAIN 3: ANALYSIS AND FINDINGS**			
**Data analysis**			
Number of data coders	24	How many data coders coded the data?	6
Description of coding tree	25	Did authors provide a description of the coding tree?	14
Derivation of themes	26	Were themes identified in advance or derived from the data?	14
Software	27	What software, if	6

		applicable, was used to manage the data?	
**Reporting**			
Participant checking	28	Did participants provide feedback on the findings?	7
Quotations presented	29	Were participant quotations presented to illustrate the themes/findings? Was each quotation identified? e.g. participant number	9-14
Data and findings consistent	30	Was there consistency between the data presented and the findings?	9
Clarity of major themes	31	Were major themes clearly presented in the findings?	16
Clarity of minor themes	32	Is there a description of diverse cases or discussion of minor themes?	16

Developed from: Tong, A.; Sainsbury, P.; Craig, J. Consolidated criteria for reporting qualitative research (COREQ): A 32-item checklist for interviews and focus groupsInt. J. Qual. Health Care 2007, 19, 349–357 [36].
